# Melatonin added to freezing diluent improves canine (Bulldog) sperm cryosurvival

**DOI:** 10.1530/RAF-20-0022

**Published:** 2020-11-04

**Authors:** Julio A Martinez-Rodriguez, Francisco J Carbajal, Rocio Martinez-De-Anda, Alicia Alcantar-Rodriguez, Alfredo Medrano

**Affiliations:** 1 Multidisciplinary Research Unit (L2), Faculty of Superior Studies Cuautitlan, National Autonomous University of Mexico, Cuautitlan Izcalli, Mexico

**Keywords:** Bulldog semen, cryopreservation, melatonin, antioxidants

## Abstract

Cryopreservation compromises the capacity of sperm fertilizing due to a series of alterations in the structure and physiology of the sperm. The use of antioxidants, such as melatonin, added to freezing media, may help to reduce sperm cryoinjury. To test the effect of melatonin on Bulldog (*Canis lupus familiaris*) sperm cryosurvival, spermatozoa were diluted in a standard freezing medium and cooled to 5°C. Then, more freezing medium was added to obtain 200 × 10^6^ cells/mL, and 5% glycerol. Diluted spermatozoa were treated with melatonin (0.0, 0.0005, 0.002, and 0.0035 mol/L), and packaged in 0.25 mL straws, which were further cooled to −5°C before freezing in liquid nitrogen. Thawing was carried out at 70°C for 5 s, and the progressive motility, viability, plasma membrane integrity, acrosome integrity, capacitation status, and plasma membrane fluidity of the spermatozoa (at 37°C) were assessed. Data were analyzed using ANOVA to detect differences between the melatonin doses. There were statistical differences (*P* < 0.05) in the percentage of sperm having hyper-fluid membranes, intact acrosome, capacitated acrosome-intact, and acrosome-reacted. The values for the high melatonin doses (0.002 and 0.0035 mol/L) were better than for the low melatonin doses (0.0 and 0.0005 mol/L). In conclusion, 0.002 and 0.0035 mol/L of melatonin improved the cryosurvival of sperm from male bulldogs.

**Lay summary:**

Preservation of sperm by freezing enables breeding of individuals geographically separated; protocols for the dog may be used to preserve the semen from threatened wild canids. To improve fertility of female dogs that become pregnant with frozen and then defrosted sperm, these cells must survive that process which can be damaging whilst keeping their ability to fertilize. Antioxidants are substances capable of retarding or preventing the oxidation of any oxidizing substrate such as lipids, proteins, and DNA, which are structural compounds of the sperm. The use of antioxidants, added to freezing media, may provide the sperm the capacity to neutralize oxidative compounds, such as reactive oxygen species, produced during the freezing and thawing process. In this work we tested different levels of melatonin, a natural antioxidant, on dog (English Bulldog) sperm survival and quality after freezing. We found that adding melatonin to the freezing media improved sperm quality after thawing.

## Introduction

Artificial insemination (AI) employing frozen-thawed spermatozoa represents a practical tool for dog breeding, especially for breeds such as the English Bulldog. Usually, males of this breed are unable to mate. Therefore, many litters are conceived by AI. Despite this, English bulldogs are a beloved breed in many countries, and their semen serves as a model to develop cryopreservation protocols for dog spermatozoa.

Freeze–thawing spermatozoa irreversibly impair the structure and function of the sperm (Watson 1995). During cryopreservation, the lipids that comprise the plasma membrane suffer the temperature-associated phenomenon known as a phase transition. As the temperature decreases, the lipids progressively change from a liquid-crystalline phase to a gel phase (Hazel 1995), and thus the plasma membrane becomes rigid. Most lipids undergo this change at temperatures above zero degrees (Watson 1995). However, there is evidence that some additional phase transitions could occur below zero degrees (Crowe *et al.* 1989). In addition, freezing affects the physical state of membrane lipids due to changes in the hydration level. The membrane phase transition may have different effects on the membrane conformational disorder in the frozen state when the ice nucleation occurs at different subzero temperatures (Balasubramanian *et al.* 2009).

Many approaches have been used to reduce cryodamage. We have tested the effect of different pre-freeze cooling temperatures on boar (Garzon-Perez *et al.* 2010), horse (Contreras-Mendez & Medrano 2016), and dog spermatozoa (Alcantar-Rodriguez & Medrano 2017, Ortega-Morales *et al.* 2019). Another approach we have tested is the use of melatonin (MLT) as an antioxidant in cooling/freezing media for the conservation of ram (Cano-Suarez *et al.* 2013), goat buck (Moazzami *et al.* 2014), and boar spermatozoa (Martinez-Hernandez *et al.* 2018). MLT improved the quality of ram spermatozoa, but those of goat buck and boar spermatozoa were not affected. MLT has also been tested for the preservation of spermatozoa from several species such as humans, horses, bulls, and buffalos with varying results (for review see, Medrano *et al.* 2017). The protective role of MLT in sperm preservation is based on its antioxidant and anti-apoptotic properties that may either maintain or improve sperm quality throughout that process (Reiter *et al.* 2007). It has been argued that the MLT action is targeted via the mitochondria by reducing oxygen consumption and, thus, reactive oxygen species (ROS) production (Lopez *et al.* 2009). The actions of MLT on sperm functionality may be mediated by specific receptors such as MT_1_ and MT_2_, which have been identified in several animal species, including dogs (González-Arto *et al.* 2016).

However, the effectiveness of MLT seems to depend on the dose at which it is employed. Depending on the animal species, the best dose for improving the sperm quality and fertile capacity ranges from 0.00001 mM to 0.003 mol/L (for review see, Medrano *et al.* 2017). High doses of MLT may reduce the sperm fertilizing potential after cryopreservation. Since ROS production is excessively neutralized, oxidative phosphorylation may be inhibited, and consequently, the sperm motility and viability reduced (Souza *et al.* 2016).

These experimental approaches are directed to reduce the occurrence of severe changes in the plasma membrane fluidity (pre-freeze cooling to subzero temperatures) and oxidative stress (antioxidants) during cryopreservation. This study aimed to test the effect of pre-freeze cooling to −5°C and MLT on dog sperm cryosurvival.

## Materials and methods

All experiments complied with the Institutional Subcommittee for Care of Animals in Experimentation from the National Autonomous University of Mexico (Subcomité Institucional para el Cuidado de Animales en Experimentación, Universidad Nacional Autónoma de Mexico). This work was carried out at the Unit of Multidisciplinary Research, Laboratory of Animal Reproduction, from the National Autonomous University of Mexico (Faculty of Superior Studies Cuautitlan).

### Animals

Ejaculates (*n* = 8, two from each male) from 4 English bulldogs (2–6 years old), collected throughout June 2017, were employed in a preliminary experiment. Ejaculates (*n* = 24, three from each male) from 8 English bulldogs (3–7 years old), collected from June to November 2018, were employed in the first experiment.

### Semen collection and assessment

Semen was collected by digital stimulation (the whole ejaculate) once a week from each male. Immediately after collection, the semen was left for 30 min at approximately 23°C (room temperature) to adjust. Then, it was macroscopically assessed for volume, color, appearance, and presence of strange particles (blood, hair and pus). Immediately after centrifugation and resuspension in a standard freezing medium (see: semen processing), a small sample (200 μL) of diluted spermatozoa was further diluted in phosphate-buffered solution (PBS; 1:5, v:v) at 37°C in a water bath, mixed, left for 10 min to equilibrate, and microscopically assessed. Only ejaculates showing at least 80% progressive motility and viability (SYBR14/PI) were included. The microscopic assessment was carried out as follows:

Progressive motility (percentage of cells showing forward movement) was subjectively assessed under light microscopy (Leica DMLS) using the 20× objective. A smear stained by eosin/nigrosine was employed to assess normal/abnormal spermatozoa (Barth & Oko 1989) under light microscopy using the 100× objective, and 200 cells were counted for each determination.

The sperm plasma membrane integrity was assessed by fluorescent staining using SYBR14/propidium iodide (PI) (L7011 Invitrogen). Moreover, 50 μL of diluted spermatozoa (in PBS 1 + 5, v/v) was added to 5 μL of SYBR14 (100 nmol/L) and mixed. Immediately after this, 5 μL of PI (12 μmol/L) was added and mixed again for 10 s. Finally, 5 μL of glutaraldehyde (0.4%) was promptly added to immobilize the spermatozoa (Garner & Johnson 1995). The percentage of live cells (green color: SYBR14-positive and PI-negative) was calculated after counting 200 spermatozoa under fluorescence microscopy (Leica DMLS) using the 100× objective.

Acrosome integrity was assessed by fluorescent lectins. Diluted sperm were smeared on a slide and air-dried, and the cells were permeabilized in alcohol for 60 min. Then, 50 μL of PSA-FITC lectin (L0770; Sigma) was spread on a slide that was left in the dark for 10 min. Immediately after, the slide was gently washed with distilled water and air-dried (Medrano *et al.* 2009). One drop of an antifade solution (DABCO 0.220 mol/L in glycerol/PBS, D-2522; Sigma) was placed on the slide, and a coverslip was positioned on top. The percentage of cells showing a smooth and well-defined acrosome was calculated after counting 200 spermatozoa under fluorescence microscopy using the 100× objective.

Sperm capacitation status was assessed using the chlortetracycline (CTC) assay as follows: 100 μL of diluted sperm (in PBS 1 + 5, v/v) was added to 100 μL of CTC solution (pH 7.8), mixed for 30 s, and 20 μL of glutaraldehyde (0.2%) was added to immobilize the sperm (Green & Watson 2001). CTC-stained sperm were mixed (1:1, v/v) with an antifade solution on a slide, and a coverslip was positioned on top. The percentage of cells showing any of the CTC patterns: F, with uniform fluorescence over the whole head (non-capacitated acrosome-intact spermatozoa); B, with a fluorescence-free band in the post-acrosomal region (capacitated acrosome-intact spermatozoa), or AR, with almost no fluorescence over the whole head except for a band of fluorescence in the equatorial segment (acrosome-reacted spermatozoa), were calculated after counting 200 spermatozoa under fluorescence microscopy using the 100× objective.

To assess the sperm plasma membrane fluidity, a merocyanine 540 assay was carried out as follows: a stock solution of merocyanine (0.005 mol/L) in dimethyl sulfoxide (DMSO; 154938; Sigma) was prepared and stored at room temperature (23°C), protected from light until use. Then, a work solution of merocyanine (0.000004 mol/L) in PBS (495 μL PBS + 5 μL merocyanine (0.005 mol/L) in DMSO) was freshly prepared (Harrison *et al.* 1996). Afterwards, 140 μL of sperm in PBS was added to 10 μL of the merocyanine working solution, mixed, and left for 1 min to interact. Then, 22 μL of glutaraldehyde (0.4%) was added to fix the sperm. One drop of this mix and one drop of the antifade solution (DABCO 0.220 mol/L in glycerol/PBS) were placed on a warm glass slide, and a glass cover slide was positioned on top. Gentle pressure was applied over the cover slide, with the aid of absorbent paper to eliminate the excess liquid. The percentage of cells showing either of the merocyanine patterns: opaque (low fluidity) or brilliant (high fluidity-high-binding cells) were calculated after counting 200 spermatozoa under fluorescence microscopy (Leica DMLS) using the 100× objective.

Not all variables were measured for all experiments. However, each stage indicates which of them were assessed.

### Semen processing

The semen was centrifuged at 750 ***g*** for 10 min, the supernatant was removed, and the cell pellet was resuspended with an egg yolk/tris-based freezing medium (EYT) containing 3% glycerol (v/v) (Peña & Linde Forsberg 2000) to obtain a concentration of 400 × 10^6^ cells/mL. These procedures were performed at about 23°C (room temperature). The sperm concentration was estimated from a 1:200 dilution (semen: formaldehyde saline solution, v/v) with the aid of a Neubauer chamber using the 40x objective.

### Cooling and freezing of spermatozoa

Sperm diluted in EYT (3% glycerol) were cooled from room temperature to 5°C for approximately 2 h (0.15°C/min). Then, EYT medium containing 7% glycerol (v/v) was slowly added in three equal parts with 10 min periods between each to obtain a final concentration of 200 × 10^6^ cells/mL and 5% glycerol. Then, the diluted spermatozoa were divided into aliquots and MLT was added as follows:

Preliminary experiment: (i) 0.0, (ii) 0.001, and (iii) 0.002 mol/L
First experiment: (i) 0.0, (ii) 0.0005, (iii) 0.002, and (iv) 0.0035 mol/L

**Figure 1:**
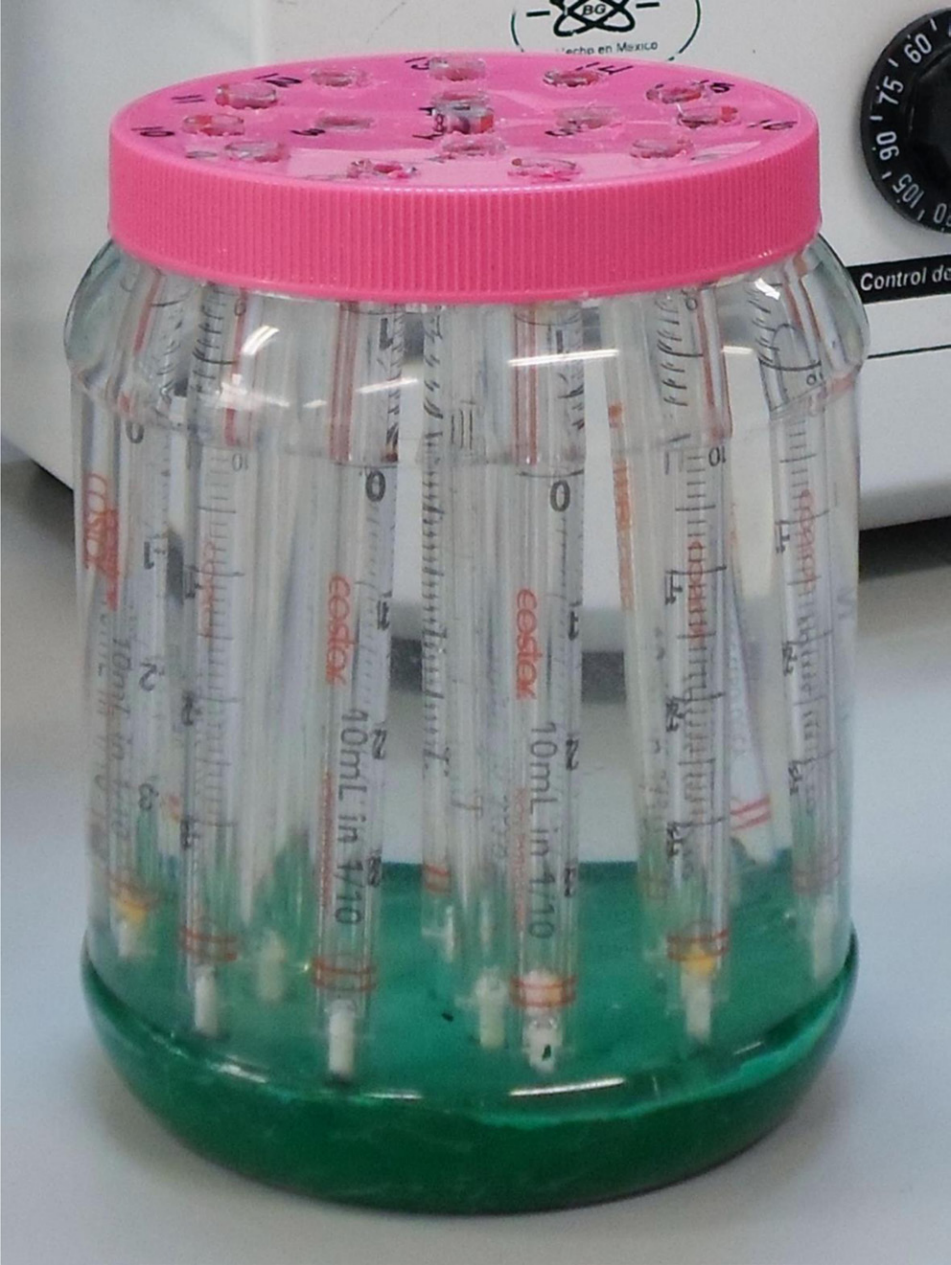
The special recipient containing saline solution (NaCl 10%, w/v) employed to cool the straws from 5°C to −5°C; each straw (*n* = 16) was inserted inside one of the plastic cylinders.

A mix of DMSO + PBS (1 + 9, v/v) was added to the control group (i.e. 0.0 mol/L of MLT) so that all treatments contained the same amount of DMSO (used to dissolve MLT). Then, the diluted spermatozoa were packaged in 0.5 mL (preliminary experiment) or 0.25 mL (first experiment) plastic straws, which were sealed with polyvinyl alcohol. The straws were placed in a special recipient containing a saline solution (NaCl 10%, w/v) at 5°C. Each straw was positioned inside a plastic cylinder with a diameter slightly larger than the straw. One end of these cylinders was sealed and fixed to the bottom of the recipient using plasticine, while the other end was opened and stood above water (Fig. 1). In this way, the straws were maintained in a vertical position, separated from each other, and dried. The straws were further cooled to −5°C (0.12°C/min) by immersing the special recipient in crushed saline ice (NaCl 10%, w/v) at −12°C. This method has been previously validated in our laboratory (Alcantar-Rodriguez & Medrano 2017). The temperature was monitored with the aid of a thermocouple (HANNA Instruments, USA) positioned inside a straw containing EYT (5% glycerol). When the straw reached −5°C, they were exposed to nitrogen vapors, 4 cm above liquid nitrogen for 15 min, and then immersed in liquid nitrogen and stored in a Dewar until required.

### Thawing of spermatozoa

The straws (12 per ejaculate, three per treatment) were thawed at 37°C for 30 s (preliminary experiment) or 70°C for 5 s (first experiment) in a water bath. The content was immediately poured into dry tubes located in another water bath at 37°C. After 10 min, the sperm were assessed as described previously.

### Experimental design

To assess the effect of MLT on the gross measures of sperm quality, a preliminary experiment was carried out to test the effect of 0.0, 0.001, and 0.002 mol/L of MLT on the cryosurvival of spermatozoa from English bulldogs (four males (replicates), two ejaculates per male). The semen was collected at the laboratory, assessed, and processed as previously mentioned. Each ejaculate was processed separately and subjected to all the treatments (aliquots). The diluted spermatozoa were cooled to either +5°C or −5°C, and frozen–thawed as previously mentioned. The progressive motility, plasma membrane integrity, capacitation status, and acrosome integrity were assessed before and after cryopreservation. This experiment was replicated four times.

In the first experiment, the effects of 0.0, 0.0005, 0.0020, and 0.0035 mol/L of MLT were tested on the cryosurvival of spermatozoa from English bulldogs (eight males (replicates), three ejaculates per male). The semen was collected at the laboratory, assessed, and processed as mentioned. Each ejaculate was processed separately and subjected to all treatments (aliquots). The diluted spermatozoa were cooled to −5°C, and freeze–thawed as previously mentioned.

The progressive motility, plasma membrane integrity, plasma membrane fluidity, capacitation status, and acrosome integrity were assessed before and after cryopreservation. This experiment was replicated eight times.

**Figure 2:**
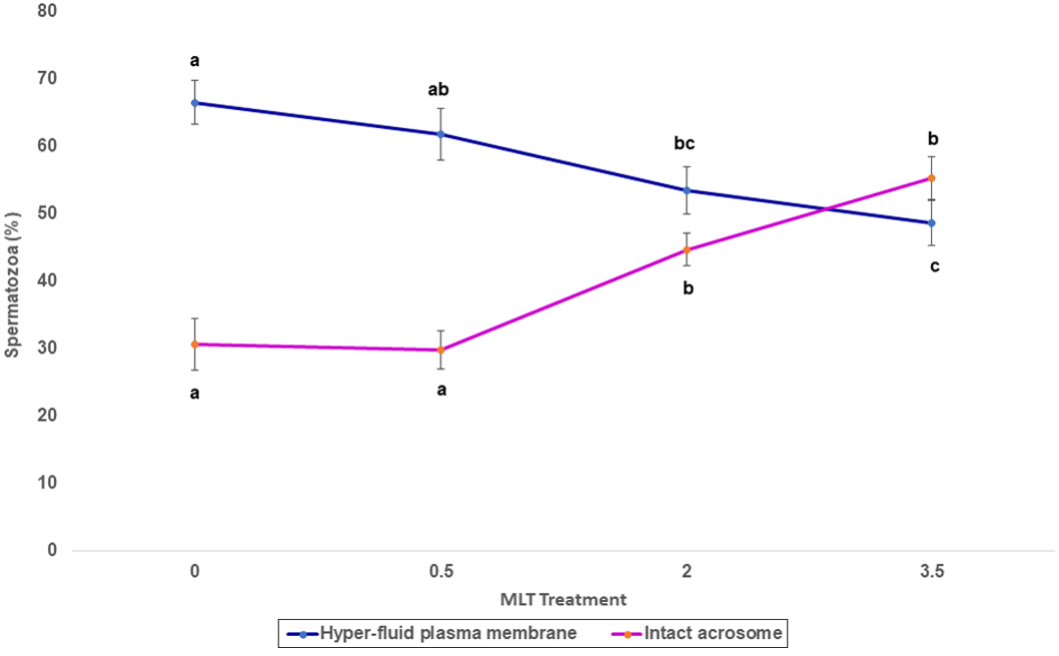
Hyper-fluid plasma membranes (%) and intact acrosomes (%) for each for each of the treatments of melatonin (MLT). 0, 0.0; 0.5, 0.0005; 2, 0.002; 3.5, 0.0035 mol/L. Values are means ± s.e.m. Different letters within each line indicate significant differences (*P* < 0.05).

### Statistical analysis

Sperm quality data from the fresh semen were analyzed using descriptive statistics only. Data regarding the sperm quality from the cooling treatments of the preliminary experiment (i.e. +5 vs −5°C) were analyzed using the paired T test; data from the MLT treatments and individual dogs were pooled for each cooling temperature. For the ANOVA of cooling temperatures x MLT doses, an individual dog was a fixed effect. The sperm quality data from the (i) cooling temperatures × MLT treatments (preliminary experiment), and (ii) MLT treatments (first experiment) were arcsine transformed to normalize the data before the ANOVA (Snedecor & Cochran 1989). The general linear model procedure from SPSS version 15.0 (2006) was used in the analysis. Effects included in the model of the first experiment were the treatments (0.0, 0.0005, 0.0020, 0.0035 mol/L MLT), and interaction dog × treatment. Results were expressed as mean ± s.e.m. Values were considered to be significant at *P* < 0.05.

## Results

In the preliminary experiment, there were significant differences (*P* < 0.05) between the cooling temperatures in the percentages of non-capacitated, acrosome-intact spermatozoa (Pattern F, CTC assay): 17.9 ± 3.50% (+5°C) vs 30.8 ± 6.32% (−5°C); capacitated, acrosome-intact spermatozoa (Pattern B, CTC assay): 69.9 ± 2.53% (+5°C) vs 59.4 ± 4.71% (−5°); and acrosome-intact spermatozoa (PSA-FITC lectin): 58.9 ± 5.56% (+5°C) vs 72.8 ± 4.29% (−5°C; Table 1). In contrast, there were no significant differences in any of the assessed variables between the MLT doses or between cooling and MLT (Table 2).

**Table 1:** Effect of cooling to different target temperatures on English Bulldog sperm cryosurvival. Values are presented as means ± s.e.m.

Pre-freeze cooling	Progressive motility (%)	Plasma membrane integrity (%)	Capacitation status (CTC patterns)			Acrosome integrity (%)
			F (%)	B (%)	AR (%)	
+5°C	24.9 ± 3.34	28.3 ± 2.48	17.9 ± 3.50a	69.9 ± 2.53a	12.0 ± 1.59	58.9 ± 5.56a
−5°C	22.7 ± 3.25	33.2 ± 2.67	30.8 ± 6.32b	59.4 ± 4.71b	10.0 ± 2.15	72.8 ± 4.29b

Different letters in columns indicate significant differences (*P* < 0.05).

**Table 2:** Effect of cooling to different target temperatures and melatonin (MLT) on English Bulldog sperm cryosurvival. Values are presented as means ± s.e.m.

Pre-freeze cooling/MLT	Progressive motility (%)	Plasma membrane integrity (%)	Capacitation status (CTC patterns)			Acrosome integrity (%)
			F (%)	B (%)	AR (%)	
+5°C						
0	26.4 ± 8.48	24.6 ± 5.86	20.7 ± 11.05	71.8 ± 9.71	7.5 ± 3.88	75.5 ± 2.50
1	22.0 ± 7.02	35.7 ± 5.15	21.8 ± 7.99	69.5 ± 5.63	8.7 ± 2.40	50.3 ± 25.25
2	24.8 ± 6.59	21.9 ± 2.38	25.3 ± 12.05	64.0 ± 7.57	9.7 ± 4.28	64.7 ± 19.91
−5°C						
0	24.8 ± 7.40	40.7 ± 4.44	43.2 ± 18.39	50.8 ± 14.13	6.0 ± 4.54	88.0 ± 7.00
1	19.0 ± 6.03	31.7 ± 4.78	49.0 ± 16.00	47.3 ± 15.25	3.8 ± 0.75	82.3 ± 11.75
2	17.5 ± 5.88	31.1 ± 6.20	42.3 ± 19.75	56.3 ± 18.75	1.5 ± 1.00	82.3 ± 5.33

There were no differences between treatments (cooling x melatonin). MLT: 0, 0.0; 1, 0.001; 2, 0.002 mol/L.

In the first experiment, there were significant differences (*P* < 0.05) in the percentages of sperm with hyper-fluid membranes (MC540 brilliant pattern) between the 0.0 and either 0.002 or 0.0035 mol/L of MLT (Fig. 2).

In addition, there were significant differences (*P* < 0.05) in the percentages of sperm with intact acrosomes between the low (0.0 and 0.0005 mol/L) and high (0.002 and 0.0035 mol/L) MLT treatments. The values of the high MLT doses were higher than those of the low MLT treatments (Fig. 2).

There were significant differences (*P* < 0.05) in the percentages of capacitated acrosome-intact spermatozoa (Pattern B, CTC assay) between the low and high MLT treatments. There were also significant differences (*P* < 0.05) in the percentages of acrosome-reacted spermatozoa (Pattern AR, CTC assay) between the low and high MLT treatments (Table 3).

**Table 3:** Effect of melatonin (MLT) on English Bulldog sperm cryosurvival. Values are presented as means ± s.e.m.

MLT	Progressive motility (%)	Plasma membrane integrity (%)	Hyper-fluid plasma membrane (%)	Capacitation status (CTC patterns)			Acrosome integrity (%)
				F (%)	B (%)	AR (%)	
0	42.7 ± 4.63	33.2 ± 3.42	66.5 ± 3.20a	21.9 ± 2.23	34.4 ± 2.29a	45.6 ± 2.88a	30.6 ± 3.81a
0.5	36.5 ± 4.56	36.1 ± 3.52	61.8 ± 3.84ab	22.9 ± 1.55	40.4 ± 1.83a	36.8 ± 2.41a	29.8 ± 2.84a
2.0	41.3 ± 4.21	42.1 ± 2.81	53.4 ± 3.53bc	21.8 ± 1.24	51.3 ± 2.35b	26.2 ± 2.43b	44.7 ± 2.43b
3.5	45.1 ± 4.15	42.4 ± 2.85	48.6 ± 3.33c	21.7 ± 1.65	52.9 ± 2.45b	26.1 ± 2.64b	55.2 ± 3.16b

Values are means ± s.e.m. Different letters in columns indicate significant differences (*P* < 0.05). MLT: 0, 0.0; 0.5, 0.0005; 2, 0.002; 3.5, 0.0035 mol/L.

There were significant differences (*P* < 0.05) between some dogs (regardless of MLT treatment) in sperm motility: male 1 had the highest (61.5 ± 4.12%) and males 4, 6, and 8 the smallest (33.5 ± 6.04%, 35.8 ± 7.04%, 26.5 ± 3.63%, respectively). Also, there were significant differences (*P* < 0.05) in sperm plasma membrane integrity: males 1 and 2 displayed the highest (50.2 ± 5.07% and 44.9 ± 5.61%, respectively) and male 8 the smallest (24.6 ± 4.37%) percentages (Table 4).

**Table 4:** Sperm cryosurvival from different English Bulldog males regardless MLT treatments. Values are presented as means ± s.e.m.

Male	Progressive motility (%)	Plasma membrane integrity (%)	Hyper-fluid plasma membrane (%)	Capacitation status (CTC patterns)			Acrosome integrity (%)
				F (%)	B (%)	AR (%)	
1	61.5 ± 4.12a	50.2 ± 5.07a	59.7 ± 5.79	21.3 ± 2.18	47.8 ± 3.27	29.3 ± 4.20	36.1 ± 5.44
2	44.5 ± 7.61ab	44.9 ± 5.61a	54.1 ± 4.00	22.6 ± 1.67	45.5 ± 3.96	32.8 ± 3.98	32.5 ± 4.92
3	36.6 ± 5.55ab	38.9 ± 4.41ab	57.7 ± 5.95	23.1 ± 3.07	48.3 ± 3.21	32.5 ± 4.54	44.1 ± 3.76
4	33.5 ± 6.04bc	34.8 ± 3.73ab	53.3 ± 7.70	24.1 ± 2.14	46.8 ± 3.48	29.0 ± 3.29	39.5 ± 5.30
5	47.1 ± 4.86abc	39.5 ± 3.87ab	61.0 ± 5.07	24.4 ± 2.37	46.5 ± 4.22	29.1 ± 3.65	44.0 ± 5.33
6	35.8 ± 7.04bc	39.7 ± 2.78ab	63.6 ± 3.45	20.5 ± 3.06	36.8 ± 3.35	43.7 ± 5.26	28.8 ± 5.30
7	45.8 ± 5.31ac	35.1 ± 3.26ab	45.3 ± 5.06	23.5 ± 2.38	43.8 ± 2.97	32.7 ± 2.95	45.4 ± 6.18
8	26.5 ± 3.63bc	24.6 ± 4.37b	66.0 ± 1.91	17.2 ± 1.77	42.5 ± 5.59	40.3 ± 5.22	50.1 ± 4.23

Different letters in columns indicate significant differences (*P* < 0.05).

Regarding differences in sperm quality due to MLT treatments in the same dog, sperm from some males had higher (acrosome integrity, capacitated acrosome-intact) or smaller (plasma membrane hyper-fluidity, acrosome-reacted) values in some variables when high levels of MLT were added (Table 5). In contrast, the sperm quality from other males was independent of MLT treatments.

**Table 5:** Characteristics of frozen–thawed spermatozoa from eight individual Bulldog males under the effect of melatonin (MLT). Values are presented as means ± s.e.m.

Dogs/MLT treatment	Progressive motility (%)	Viability (%)	Hyperfluid membranes (%)	Intact acrosomes (%)	Capacitation status (%)		
					F	B	AR
Male 1							
0.0	68.3 ± 4.4	40.0 ± 10.4	72.8 ± 4.6	19.0 ± 3.2a	21.3 ± 7.0	38.7 ± 9.4	40.0 ± 11.6
0.5	61.0 ± 10.7	49.0 ± 13.7	68.7 ± 10.0	22.7 ± 6.7ab	21.3 ± 4.7	44.0 ± 2.3	34. 7 ± 3.7
2.0	51.7 ± 10.1	56.2 ± 12.6	52.5 ± 13.8	43.0 ± 5.6bc	25.7 ± 2.3	52.7 ± 4.1	15.7 ± 1.2
3.5	65.0 ± 7.6	55.5 ± 6.3	44.7 ± 12.7	59.7 ± 5.1c	17.0 ± 3.2	56.0 ± 5.6	27.0 ± 8.5
Male 2							
0.0	39.0 ± 19.3	41.5 ± 14.0	62.3 ± 6.7	19.5 ± 4.0	17.3 ± 2.9	31.0 ± 2.7a	51.7 ± 2.2a
0.5	38.3 ± 19.8	41.5 ± 20.1	58.7 ± 13.0	23.2 ± 4.5	28.0 ± 3.6	35.0 ± 2.9a	37.0 ± 2.5b
2.0	49.0 ± 15.4	51.8 ± 4.8	50.5 ± 5.8	41.8 ± 7.1	25.3 ± 1.9	57.7 ± 2.2b	20.3 ± 2.3c
3.5	51.7 ± 14.3	44.8 ± 5.8	45.0 ± 3.6	45.5 ± 13.8	19.7 ± 0.3	58.3 ± 2.9b	22.0 ± 3.1c
Male 3							
0.0	42.3 ± 13.8	24.2 ± 7.7	67.0 ± 11.9	31.8 ± 3.2a	25.7 ± 12.7	39.3 ± 5.6a	50.3 ± 7.5a
0.5	27.3 ± 8.2	36.8 ± 6.7	69.3 ± 12.1	34.2 ± 2.8a	22.7 ± 4.7	40.3 ± 5.5a	37.0 ± 9.1ab
2.0	33.3 ± 8.8	44.0 ± 8.9	49.8 ± 11.1	50.8 ± 3.9b	22.7 ± 3.2	54.0 ± 2.1ab	23.3 ± 4.1b
3.5	43.3 ± 15.9	50.5 ± 7.3	44.7 ± 11.8	59.5 ± 3.3b	21.3 ± 3.2	59.3 ± 3.5b	19.3 ± 0.9b
Male 4							
0.0	27.0 ±10.5	22.7 ± 8.2	52.3 ± 19.5	27.8 ± 9.1	25.0 ± 7.9	39.0 ± 8.0	36.0 ± 6.1
0.5	35.3 ± 21.5	37.5 ± 7.5	52.3 ± 19.2	28.7 ± 8.1	27.0 ± 4.6	41.7 ± 7.3	30.7 ± 10.3
2.0	35.0 ±12.6	34.0 ± 4.0	53.3 ± 19.4	48.0 ± 11.0	20.0 ± 1.5	52.3 ± 3.9	27.7 ± 2.9
3.5	36.7 ± 6.7	44.8 ± 6.1	55.0 ± 12.2	53.7 ± 9.4	24.3 ± 1.2	54.0 ± 6.9	21.7 ± 5.8
Male 5							
0.0	48.0 ± 11.1	43.8 ± 10.6	73.5 ± 4.4	36.2 ± 11.3	25.7 ± 5.6	28.3 ± 4.6a	46.0 ± 4.2a
0.5	38.3 ± 8.8	27.2 ± 2.4	68.2 ± 3.4	32.2 ± 9.2	30.3 ± 5.4	41.7 ± 4.4ab	28.0 ± 5.9ab
2.0	47.3 ± 13.2	39.5 ± 8.5	54.8 ± 13.8	42.8 ± 7.8	17.7 ± 1.5	60.0 ± 3.5b	22.3 ± 2.9b
3.5	54.7 ± 8.7	47.7 ± 4.3	47.5 ± 11.5	64.7 ± 6.1	24.0 ± 4.6	56.0 ± 5.0b	20.0 ± 5.1b
Male 6							
0.0	41.0 ± 18.6	39.2 ± 2.7	75.8 ± 3.6a	21.3 ± 10.9	19.7 ± 6.8	32.0 ± 5.1	48.3 ± 11.7
0.5	31.0 ± 9.7	47.3 ± 7.4	67.2 ± 5.2ab	21.3 ± 9.1	14.7 ± 0.9	39.3 ± 4.8	47.7 ± 5.0
2.0	43.0 ± 22.1	39.7 ± 5.2	60.8 ± 5.7ab	34.0 ± 9.5	23.0 ± 7.4	39.3 ± 10.1	34.3 ± 12.9
3.5	28.0 ± 9.1	32.7 ± 5.2	50.7 ± 4.5b	38.3 ± 14.6	24.7 ± 8.7	36.7 ± 8.8	44.3 ± 14.8
Male 7							
0.0	45.0 ± 9.0	33.0 ± 2.3	60.5 ± 9.8	36.0 ± 19.3	25.3 ± 5.3	37.3 ± 1.3	37.3 ± 4.8
0.5	39.3 ± 14.6	33.2 ± 1.0	41.3 ± 12.1	38.5 ± 14.5	20.7 ± 4.3	45.3 ± 7.4	34.0 ± 6.7
2.0	45.7 ± 8.1	39.7 ± 6.1	41.5 ± 6.0	44.5 ± 5.8	23.7 ± 3.2	43.3 ± 8.8	33.0 ± 9.1
3.5	53.3 ± 14.5	34.5 ± 13.3	38.0 ± 11.3	62.7 ± 2.4	24.3 ± 7.9	49.3 ± 4.7	26.3 ± 3.2
Male 8							
0.0	31.0 ± 13.6	21.2 ± 14.8	67.8 ± 3.1	53.2 ± 11.6	15.3 ± 3.7	29.7 ± 12.9	55.0 ± 13.5
0.5	21.7 ± 4.4	16.7 ± 2.2	69.0 ± 4.3	37.7 ± 7.8	18.7 ± 0.9	36.0 ± 8.3	45.3 ± 7.9
2.0	25.0 ± 5.0	31.8 ± 8.4	63.5 ± 1.3	52.3 ± 4.9	16.7 ± 3.8	50.7 ± 10.4	32.7 ± 9.6
3.5	28.3 ± 6.0	28.8 ± 7.4	63.5 ± 6.2	57.3 ± 7.9	18.0 ± 6.0	53.7 ± 12.0	28.3 ± 6.4

Different letters within males indicate significant differences (*P* < 0.05). MLT: 0, 0.0; 0.5, 0.0005; 2, 0.002; 3.5, 0.0035 mol/L.

## Discussion

In the preliminary experiment, the effects of MLT (0.0, 0.001, and 0.002 mol/L) and pre-freeze cooling to −5°C were tested on the cryosurvival of sperm from male English bulldogs. MLT produced no differences in sperm cryosurvival. However, pre-freeze cooling to −5°C improved some sperm attributes. In a similar study, using a different breed of dogs (Belgian and German Shepherds), cooling reduced the percentage of hyper-fluid membranes in comparison to the usual cooling to +5°C (Ortega-Morales *et al.* 2019). These observations support the proposed hypothesis that the cooling of spermatozoa to subzero temperatures favors sperm plasma membrane reorganization after the lipid phase transition takes place, thus avoiding an excessive increase in membrane fluidity (Watson 1995, Holt 2000). Balasubramanian *et al.* (2009) reported that ice nucleation at −6°C enables a fraction of cellular and membrane-bound water to stay in the cell and therefore promotes cell viability.

In the first experiment, different doses of MLT were tested on the cryosurvival of sperm from male English bulldogs. In general, sperm cryosurvival was significantly better with high (0.002 and 0.0035 mol/L) than low (0.0 and 0.0005 mol/L) doses of MLT. In this experiment, some modifications to the freeze–thawing protocol (i.e. packaging in 0.25 mL straws instead of 0.5 mL, thawing at 70°C instead of 37°C) were introduced in addition to the different doses of MLT.

Regarding changes in plasma membrane fluidity before and after cryopreservation, the values of hyper-fluid membranes obtained in this work (16.1% vs 57.6%, fresh *vs* frozen-thawed sperm) were similar to those reported by others: 11% vs 91% (dog sperm, Alhaider & Watson 2009), 3.1% vs 66.7% (dog sperm, Ortega­-Morales *et al.* 2019), and 11% vs 55.1% (pig sperm, Orozco *et al.* 2016). In addition, we measured that shift to compare the effect of MLT on plasma membrane fluidity.

We hypothesized that MLT and the cooling of spermatozoa to −5°C before freezing favors sperm plasma membrane reorganization after the lipid phase transition takes place, thus avoiding an excessive increase in membrane fluidity. Our results on membrane fluidity support our hypothesis that high doses (0.002 and 0.0035 mol/L) of MLT significantly reduce the percentage of sperm with hyper-fluid membranes after freeze–thawing. However, similar results were obtained in dog sperm by the mere effect of pre-freeze cooling (Ortega-Morales *et al.* 2019). We argue that because the difference in the percentage of hyper-fluid membranes due to MLT is larger than that of cooling to −5°C alone, there is an additive effect of MLT and cooling.

The protective role of MLT in sperm preservation is based on its antioxidant and anti-apoptotic properties that may either improve or maintain sperm quality (Reiter *et al.* 2007). There is a lack of information regarding the most appropriate doses of MLT for dog sperm cryopreservation. Varesi *et al.* (2014) found no differences in dog sperm cryosurvival (motility, acrosome integrity) when 0.001 mol/L of MLT was added to the freezing extender. In ram spermatozoa, the sperm motility, DNA integrity, intracellular concentration of ATP, and fertilization rate after freeze–thawing were higher in the sperm supplemented with 0.001 mol/L of MLT than with smaller or larger doses (Succu *et al.* 2011). In bulls, 0.002 and 0.003 mol/L of MLT improved sperm cryosurvival in terms of the sperm motility, viability, plasma membrane integrity, normal spermatozoa, superoxide dismutase, and catalase activity (Ashrafi *et al.* 2013). In buffalo, the sperm motility, plasma membrane integrity, acrosome integrity, and conception rate after freeze–thawing were higher in the sperm supplemented with 0.0001 and 0.00025 mol/L of MLT than in the non-supplemented sperm (El-Raey *et al.* 2015). In horses, the plasma membrane integrity, acrosome integrity, and high mitochondrial activity improved in the frozen-thawed sperm supplemented with 0.001 mol/L of MLT (Lanconi *et al.* 2015). In humans, the motility, viability, and reduction of intracellular concentration of ROS were higher in the frozen-thawed sperm supplemented with 0.00001 mol/L of MLT than in those supplemented with smaller or larger doses (Karimfar *et al.* 2015). Therefore, MLT seems to be effective in a dose of species fashion. Due to this, it may be interesting to test higher (>0.0035 mol/L) doses of MLT on dog sperm cryosurvival.

MLT in high doses may reduce the potential of sperm fertilizing after cryopreservation. The sperm motility and viability are reduced due to ROS production being excessively neutralized and oxidative phosphorylation inhibited (Souza *et al.* 2016).

In conclusion, 0.002 and 0.0035 mol/L of MLT was better than the other concentrations of MLT in terms of the quality of post-thawed dog spermatozoa.

## Declaration of interest

The authors declare that there is no conflict of interest that could be perceived as prejudicing the impartiality of the research reported.

## Author contribution statement

J A M-R, F J C, and R M-D-A carried out semen collection, freeze–thawing experiments, collected data and drafted the paper. A A R and A M designed and supervised the work, analyzed data, drafted and corrected the paper.
